# Higher risk of cardiovascular mortality than cancer mortality among long-term cancer survivors

**DOI:** 10.3389/fcvm.2023.1014400

**Published:** 2023-01-25

**Authors:** Zhipeng Wang, Zeyu Fan, Lei Yang, Lifang Liu, Chao Sheng, Fengju Song, Yubei Huang, Kexin Chen

**Affiliations:** ^1^Department of Epidemiology and Biostatistics, Key Laboratory of Molecular Cancer Epidemiology, National Clinical Research Center for Cancer, Tianjin Medical University Cancer Institute and Hospital, Tianjin Medical University, Tianjin, China; ^2^Key Laboratory of Carcinogenesis and Translational Research (Ministry of Education/Beijing), Beijing Office for Cancer Prevention and Control, Peking University Cancer Hospital and Institute, Beijing, China; ^3^Department of Statistics, European Organization for Research and Treatment of Cancer, Brussels, Belgium

**Keywords:** cancer, cardiovascular deaths and mortality, cardio-oncology, long-term cancer survivors, anticancer treatment

## Abstract

**Background:**

Previous studies focused more on the short-term risk of cardiovascular (CV) death due to traumatic psychological stress after a cancer diagnosis and the acute cardiotoxicity of anticancer treatments than on the long-term risk of CV death.

**Methods:**

Time trends in the proportions of CV death (P_CV_), cancer death (P_CA_), and other causes in deaths from all causes were used to show preliminary relationships among the three causes of death in 4,806,064 patients with cancer from the Surveillance, Epidemiology, and End Results (SEER) program. Competing mortality risk curves were used to investigate when the cumulative CV mortality rate (CMR_CV_) began to outweigh the cumulative cancer mortality rate (CMR_CA_) for patients with cancer who survived for more than 10 years. Multivariable competing risk models were further used to investigate the potential factors associated with CV death.

**Results:**

For patients with cancer at all sites, the P_CV_ increased from 22.8% in the 5th year after diagnosis to 31.0% in the 10th year and 35.7% in the 20th year, while the P_CA_ decreased from 57.7% in the 5th year after diagnosis to 41.2 and 29.9% in the 10th year and 20th year, respectively. The P_CV_ outweighed the P_CA_ (34.6% vs. 34.1%) since the 15th year for patients with cancer at all sites, as early as the 9th year for patients with colorectal cancer (37.5% vs. 33.2%) and as late as the 22nd year for patients with breast cancer (33.5% vs. 30.6%). The CMR_CV_ outweighed the CMR_CA_ since the 25th year from diagnosis. Multivariate competing risk models showed that an increased risk of CV death was independently associated with older age at diagnosis [hazard ratio and 95% confidence intervals [HR (95%CI)] of 43.39 (21.33, 88.28) for ≥ 80 vs. ≤ 30 years] and local metastasis [1.07 (1.04, 1.10)] and a decreased risk among women [0.82 (0.76, 0.88)], surgery [0.90 (0.87, 0.94)], and chemotherapy [0.85 (0.81, 0.90)] among patients with cancer who survived for more than 10 years. Further analyses of patients with cancer who survived for more than 20 years and sensitivity analyses by cancer at all sites showed similar results.

**Conclusion:**

CV death gradually outweighs cancer death as survival time increases for most patients with cancer. Both the cardio-oncologist and cardio-oncology care should be involved to reduce CV deaths in long-term cancer survivors.

## Introduction

Cancer ranks as the leading cause of death and remains the primary barrier to increasing life expectancy in most global countries in the twenty-first century (1). According to recent estimates from the World Health Organization (WHO), cancer is the first or second leading cause of death below the age of 70 in 91 out of 172 countries and ranks third or fourth in another 22 countries (1). Moreover, according to a recent study on the Global Burden of Disease, cancer cases rose by 28% between 2006 and 2016 (2). This rise seems to continue due to the growing epidemic of cancer-associated risk factors, including aging, tobacco use, unhealthy diet, excess body weight, and physical inactivity. According to the most recent GLOBOCAN estimates, there would be 19.3 million new cases and almost 10 million cancer deaths worldwide in 2020 (3).

A cancer diagnosis is often associated with immediate and traumatic psychological stress, thereby increasing the risk of short-term cardiovascular (CV) death (4–6). As reported by Fang, the risk of CV death after a cancer diagnosis is 6–10 times higher than that in cancer-free people (5). However, the risk of CV death due to psychological stress began to decline rapidly 1 year after a cancer diagnosis (5, 7, 8). Therefore, several previous studies paid more attention to the immediate risks of CV death but less attention to the long-time risk of CV death. Due to the long-term cumulative cardiotoxicity of anticancer treatments, the potential long-time risk of CV death after anticancer treatments among patients with cancer has always been a great concern for both oncologists and cardiologists (9–12). Previous studies suggested the increased risk of developing CV disease (CVD) among long-term cancer survivors, including patients who were young (13) or patients older than 40 years (14), patients with colorectal cancer who survived for more than 10 years (15), and other patients with breast, prostate, or bladder cancer (16). A recent study suggested that the risk of fatal heart disease increased over time for almost cancer survivors at all sites (17). However, it is still unclear whether the long-term risk of CV mortality outweighs the cancer mortality risk for patients with cancer at all sites in a particular window of time. More importantly, as multimorbidity, that is, the simultaneous presence of multiple chronic conditions, including cancer and CVD due to shared risk factors, is becoming an increasing global health problem, and the integrated management of shared risk factors has become a global need to reduce the overall burden of multimorbidity (18–21). However, current treatment and management protocols that treat cancer as a separate disease entity are likely to ignore the long-term risk of CV death in cancer survivors.

Therefore, this study aimed to investigate the long-time risk of CV death among cancer survivors and investigate whether and when CV death outweighed cancer death for cancer survivors at different sites.

## Materials and methods

### Study cohort

A network of population-based incident tumor registries from geographically distinct regions in the USA was established as the Surveillance, Epidemiology, and End Results (SEER) program of the National Cancer Institute (22, 23). Data on patient demographics, the month and year of diagnosis, tumor characteristics, treatment utilization, and mortality from all incident cancer cases from the selected population-based cancer registries in the USA were reported uniformly and then collected by the SEER program.

The SEER 9 data, which covered 10% of the entire US population and were released in April 2018, were used (22, 24). All patients with cancer diagnosed between 1 January 1973 and 31 December 2015 in the nine original registries (San Francisco-Oakland, Connecticut, Detroit, Hawaii, Iowa, New Mexico, Seattle, Utah, and Atlanta) were included in SEER 9 (25). In the most recent SEER data, all cancers were classified into nine sites: respiratory (RESPIR), breast (BREAST), colon and rectum (COLRECT), urinary (URINARY), lymphoma of all sites and leukemia (LYMYLEUK), female genital (FEMGEN), male genital (MALEGEN), other digestive except the colon and the rectum (DIGOTHR), and all other sites except the abovementioned sites (OTHER).

Radiation treatment variables were removed from the public research database since November 2016, and chemotherapy had never been released in the research data. Therefore, radiation and chemotherapy data were available through personalized data (26). Finally, a total of 5,094,238 patients with cancer diagnosed as the first primary malignancy were initially identified. We excluded 48,820 (1.0%) patients with cancer with no clear cause of death, 67,735 (1.3%) patients with cancer loss to a follow-up, and 171,619 (3.3%) patients with cancer diagnosed in 2015 for less than a 1-year follow-up. After diagnosis, a total of 4,806,064 patients were included in the final analyses.

### Ascertainment of mortality and survival time

Data from the SEER were linked with data from the National Center for Health Statistics to determine death and its cause. Survival time, as recorded in months, started on the date of a cancer diagnosis and ended on the date of death, the last date known to be alive, or the end of a follow-up (31 December 2015), whichever happened first.

### Definition of CV death and cancer-related death

According to a previous study (13), based on the SEER Causes of Death Register and International Classification of Diseases (10th Revision, ICD10), CV deaths included deaths from heart diseases (ICD10: I00-I09, I11, I13, I20-I51), hypertension without heart disease (ICD10: I10, I12), cerebrovascular diseases (ICD10: I60-I69), atherosclerosis (ICD10: I70), and diabetes mellitus (ICD10: E10-E14). Cancer-related deaths were defined as deaths from cancer at all sites and included deaths from both primary and additional cancer sites.

### Statistical analysis

Time trends in the proportions of CV death (P_CV_), cancer death (P_CA_), and other causes in all-cause deaths were used to show preliminary relationships among the three aforementioned causes of death in patients with cancer. To investigate when the cumulative CV mortality rate (CMR_CV_) began to outweigh the cumulative cancer mortality rate (CMR_CA_) since diagnosis, cumulative incidence functions based on the Fine-Gray hypothesis were first used to calculate CMR_CV_ and CMR_CA_, respectively. Fine-Gray tests were then used to select for potential factors associated with CV death in long-time cancer survivors. Multivariable competing risk models based on the Fine-Gray tests were further used to investigate the independent factors associated with CV death (27–29). Hazard ratios and 95% confidence intervals [HR (95%CI)] were calculated based on multivariate competing risk models to measure associations between risk factors and CV death. Given the potential higher risk of arterial thromboembolic events caused by tumor thrombi among patients with LYMYLEUK (30), patients with LYMYLEUK were used as the reference group in the multivariate competing risk models.

To reduce the “dilution” effect of a short-time follow-up on the long-time risk of CV death, we selected patients with cancer who survived for more than 15 and 20 years for subgroup analysis. Moreover, to determine whether some patients with cancer never have a higher risk of CV death than cancer death in their lifetime, we further selected patients with cancer who survived for more than 25 years for a sensitivity analysis.

All analyses were performed with R software (version 4.0.3). All statistics were tested on two sides, and *p*-values of < 0.05 were considered statistically significant.

## Results

### Overall mortality rates of CV death among patients with cancer

After an average follow-up of 6.90 years, 563,848 patients with cancer died from CVD (16.99/1,000 person-years), which accounted for 17.9% of all-cause deaths (3,149,601) and 51.4% of non-cancer deaths (1,095,209). The Fine-Gray tests showed that a significantly higher overall mortality rate of CVD was found among patients with cancer who were older at diagnosis, men, widowed, African-American, patients with RESPIR, without surgery, without radiation therapy, without chemotherapy, and distant metastasis (all *p*-values < 0.001, Table 1).

**TABLE 1:** Cardiovascular mortality risk in patients with cancer by baseline characteristics.

Variable		Number of subgroup	Number of CVD death	Mean follow-up years	CVD-specific mortality rate, 1/1,000 person-years	*P*-value for Fine- Gray test
Age at diagnosis, years	≤30	177,119	2,308	13.14	0.99	<0.001
	31–39	247,533	5,863	11.51	2.06	
	41–49	522,030	21,271	9.87	4.13	
	51–59	917,621	65,447	8.10	8.80	
	61–69	1,123,669	123,222	6.91	15.87	
	71–77	1,075,716	184,835	5.09	33.77	
	≥80	742,376	160,902	2.93	73.92	
Sex	Men	2,385,471	298,205	6.03	20.71	<0.001
	Women	2,420,593	265,643	7.76	14.14	
Marital status at diagnosis	Married	2,710,794	301,105	7.61	14.60	<0.001
	Single	579,805	45,349	7.20	10.86	
	Separated	58,599	9,224	5.43	28.98	
	Divorced	360,536	30,966	6.29	13.66	
	Widowed	770,078	143,570	4.51	41.38	
	Unmarried	2,229	18	2.26	3.57	
	Unknown	324,023	33,616	7.15	14.52	
Race	White	4,045,601	489,894	7.05	17.16	<0.001
	Black	432,341	48,756	5.65	19.97	
	Indian/Alaska native	23,651	1,661	6.12	11.48	
	Asian/Pacific Islander	276,870	22,916	6.65	12.45	
	Unknown	27,601	621	7.65	2.94	
Cancer site	RESPIR	642,669	45,141	2.49	28.24	<0.001
	BREAST	786,979	96,593	10.19	12.04	
	COLRECT	529,644	93,863	6.73	26.33	
	URINARY	326,229	54,657	7.15	23.43	
	LYMYLEUK	387,832	35,673	5.95	15.47	
	FEMGEN	316,790	39,091	9.95	12.40	
	MALEGEN	641,129	111,865	8.60	20.28	
	DIGOTHR	364,418	20,575	2.19	25.84	
	OTHER	810,374	66,390	7.27	11.27	
Surgery	No	1,770,680	158,700	3.62	24.79	<0.001
	Yes	2,951,990	396,461	8.97	14.97	
	Unknown	83,394	8,687	3.53	29.49	
Radiotherapy	No	4,107,490	504,379	6.64	18.50	<0.001
	Yes	695,019	59,146	8.48	10.04	
	Unknown	3,555	323	6.84	13.28	
Chemotherapy	None/Unknown	3,712,398	513,612	7.46	18.53	<0.001
	Yes	1,093,666	50,236	5.00	9.19	
SEER historic stage	*In situ*	295,848	31,025	10.41	10.08	<0.001
	Distant	828,146	42,268	2.33	21.91	
	Localized/Regional	2,610,184	346,802	8.62	15.42	
	Unknown	1,071,886	143,753	5.30	25.32	
Total		4,806,064	563,848	6.90	16.99	

CVD, cardiovascular disease; RESPIR, respiratory; BREAST, breast; COLRECT, colon and rectum; URINARY, urinary; LYMYLEUK, lymphoma of all sites and leukemia; FEMGEN, female genital; MALEGEN, male genital; DIGOTHR, other digestive; OTHER, all other sites.

### Temporal trends in the P_CV_ and P_CA_

**FIGURE 1:**
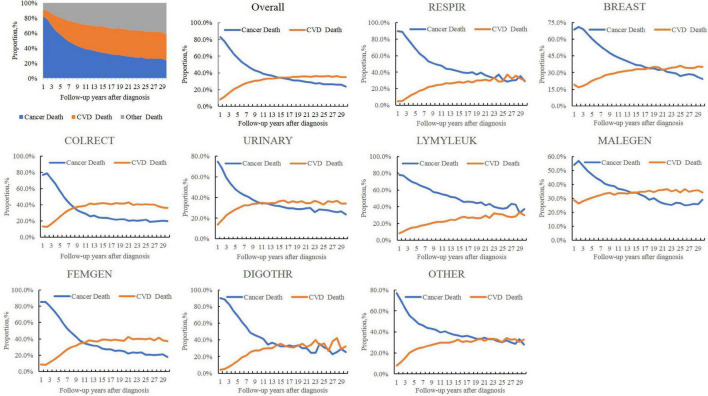
Temporal trends in the proportions of cardiovascular (CV) death (P_CV_), cancer death (P_CA_), and other causes in all-cause deaths in patients with cancer. CVD, cardiovascular disease; RESPIR, respiratory; BREAST, breast; COLRECT, colon and rectum; URINARY, urinary; LYMYLEUK, lymphoma of all sites and leukemia; FEMGEN, female genital; MALEGEN, male genital; DIGOTHR, other digestive; OTHER, all other sites.

As shown in Figure 1, for all patients with cancer, the P_CV_ increased from 22.8% in the 5th year after a cancer diagnosis, to 31.0% in 10th year, and to 35.7% in the 20th year, while the P_CA_ decreased from 57.7% in the 5th year to 41.2 and 29.9% in 10th year and 20th year, respectively. The P_CV_ outweighed the P_CA_ (34.6% vs. 34.1%) since the 15th year for all patients with cancer, as early as the 9th year for patients with colorectal cancer (37.5% vs. 33.2%) and as late as the 22nd year for patients with breast cancer (33.5% vs. 30.6%). For patients with respiratory and hematological cancers, it was not observed that the P_CV_ outweighed the P_CA_ even at a follow-up of ≥ 30 years. The same was true for patients with digestive cancers than for those with colorectal cancer and cancer at all other sites.

### Cumulative risk of cardiovascular death (CMR_CV_) and cancer death (CMR_CA_)

**FIGURE 2:**
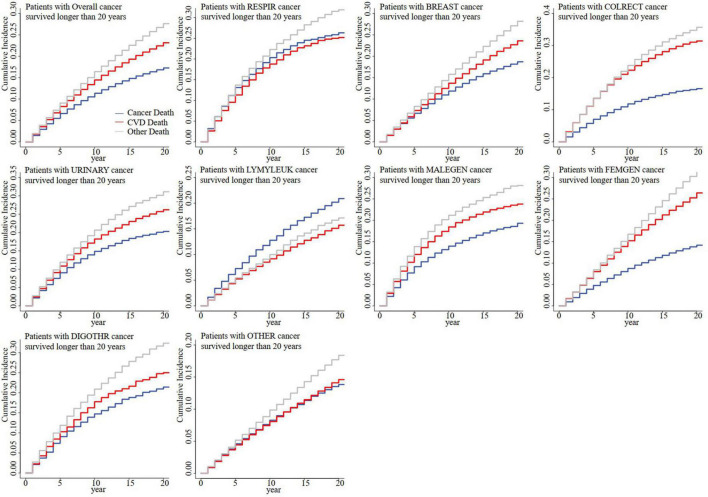
Competing mortality risk curves for patients with cancer who survived for more than 20 years by cancer sites. CVD, cardiovascular disease; RESPIR, respiratory; BREAST, breast; COLRECT, colon and rectum; URINARY, urinary; LYMYLEUK, lymphoma of all sites and leukemia; FEMGEN, female genital; MALEGEN, male genital; DIGOTHR, other digestive; OTHER, all other sites.

For all patients with cancer, the CMR_CV_ outweighed the CMR_CA_ since the 15th year for patients with cancer who survived for more than 10 years (Supplementary Figure 1), the 5th year for patients with cancer who survived for more than 15 years (Supplementary Figure 1), and the 1st year for patients with cancer who survived for more than 20 years (Figure 2). For cancer at different sites, it was observed that the CMR_CV_ finally outweighed CMR_CA_ in most patients with cancer survived for more than 10 years, except respiratory cancer, hematological cancer, digestive cancer except colorectal cancer, and cancer at all other sites (Supplementary Figure 1). For patients with cancer who survived for more than 20 years, it was only observed that CMR_CV_ finally did not outweigh CMR_CA_ in patients with respiratory and hematological cancers (Figure 2). For patients with cancer who survived for more than 15 years, it was observed that CMR_CV_ finally did not outweigh CMR_CA_ in patients with respiratory, hematological and all other types of cancers (Supplementary Figure 2). Sensitivity analyses showed that the CMR_CV_ began to outweigh CMR_CA_ in the 10th year for patients with respiratory cancer who survived for more than 25 years and in the 7th year for patients with respiratory cancer who survived for more than 30 years (Supplementary Figure 3).

### Factors associated with CV death for long-term cancer survivors

**FIGURE 3:**
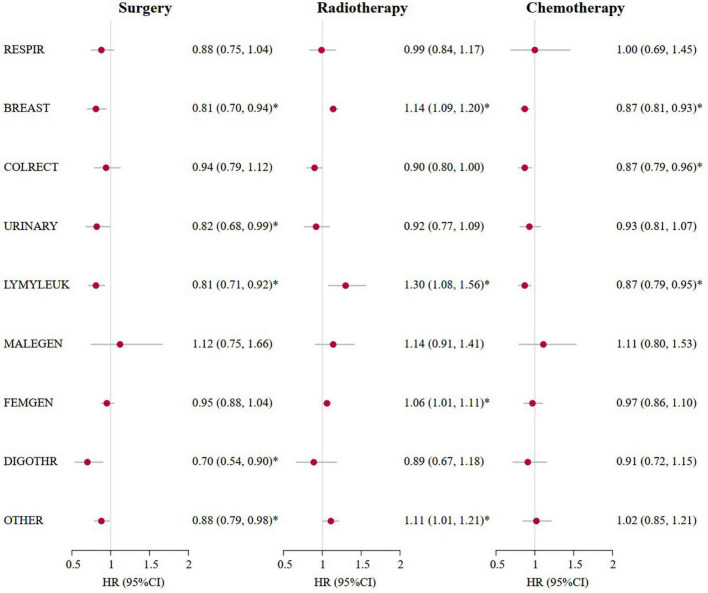
Cardiovascular mortality risk and anticancer treatments for patients with cancer who survived for more than 20 years by cancer sites. *A *p*-value of < 0.05. Marital status at diagnosis, race, calendar years at diagnosis according to 1970s, 1980s, 1990s, 2000s, 2010s, nine original registries, sex, SEER historic stage, surgery, radiotherapy and chemotherapy were adjusted in the multivariate competing risk regression models. HR (95%CI), Hazard ratio (95% confidence intervals); RESPIR, respiratory; BREAST, breast; COLRECT, colon and rectum; URINARY, urinary; LYMYLEUK, lymphoma of all sites and leukemia; FEMGEN, female genital; MALEGEN, male genital; DIGOTHR, other digestive; OTHER, all other sites.

As presented in Table 2, the multivariate competing risk models showed that an increased risk of CV death was independently associated with older age at diagnosis [HR (95%CI) of 43.39 (21.33, 88.28) for ≥ 80 years vs. ≤ 30 years] and local metastasis [1.07 (1.04, 1.10)] and a decreased risk with women [0.82 (0.76, 0.88)], surgery [0.90 (0.87, 0.94)], and chemotherapy [0.85 (0.81, 0.90)] among patients with cancer who survived for more than 10 years. Further analyses of patients with cancer who survived for more than 20 years showed similar results, while radiotherapy significantly increased the risk of CV death in long-term cancer survivors. Of all the risk factors associated with CV death, elder age at diagnosis was the most important factor. Compared with patients with cancer who survived for more than 20 years and were diagnosed at ≤ 30 years, the HRs for the risk of CV death increased from 2.07 (95%CI: 1.91–2.25) for patients who were diagnosed at 31–39 years, to 27.08 (95%CI: 21.26–34.49) for patients who were diagnosed at 71–77 years, and 40.66 (95%CI: 26.13–63.25) for patients who were diagnosed at ≥ 80 years. Sensitivity analyses by cancer sites showed similar results (Figure 3 and Supplementary Table 1).

**TABLE 2:** Cardiovascular mortality risk and its influencing factors in long-term cancer survivors*.

Variable	Groups	Patients with cancer who survived ≥ 10 years (*n* = 1,160,503)			Patients with cancer who survived ≥ 15 years (*n* = 652,029)			Patients with cancer who survived ≥ 20 years (*n* = 343,189)		
		Number of subgroups	Number of cardiovascular death	HR (95%CI)	Number of subgroups	Number of cardiovascular death	HR (95%CI)	Number of subgroups	Number of cardiovascular death	HR (95%CI)
Age at diagnosis, years	≤30	87,197	1,469	Reference	65,994	1,235	Reference	48,153	983	Reference
	31–39	110,462	3,368	2.12 (1.89, 2.37)	78,689	2,639	2.10 (1.92, 2.30)	52,347	1,944	2.07 (1.91, 2.25)
	41–49	200,749	10,840	4.16 (3.53, 4.91)	130,276	8,136	4.20 (3.74, 4.73)	78,600	5,676	4.18 (3.86, 4.53)
	51–59	277,205	28,688	8.18 (6.45, 10.38)	161,845	20,178	8.36 (7.08, 9.88)	85,736	13,098	8.38 (7.66, 9.15)
	61–69	271,128	46,748	14.36 (9.99, 20.65)	145,603	29,487	15.40 (11.70, 20.27)	63,619	15,494	16.24 (14.32, 18.42)
	71–77	173,992	46,333	27.27 (15.61, 47.66)	62,999	18,368	28.37 (17.98, 44.75)	14,137	4,626	27.08 (21.26, 34.49)
	≥80	39,770	14,386	43.39 (21.33, 88.28)	6,623	2,589	42.58 (23.55, 76.97)	597	257	40.66 (26.13, 63.25)
Sex	Men	444,613	56,090	Reference	220,643	26,382	Reference	99,120	11,505	Reference
	Women	715,890	95,742	0.82 (0.76, 0.88)	431,386	56,250	0.83 (0.78, 0.88)	244,069	30,573	0.84 (0.81, 0.87)
Cancer site	LYMYLEUK	79,645	7,666	Reference	43,846	3,965	Reference	24,381	1,995	Reference
	RESPIR	32,409	5,054	1.07 (0.98, 1.18)	15,344	2,306	1.04 (0.96, 1.13)	6,646	924	0.97 (0.89, 1.06)
	BREAST	326,879	40,587	0.98 (0.95, 1.02)	194,104	23,537	0.95 (0.90, 0.99)	104,204	12,268	0.89 (0.83, 0.95)
	COLRECT	133,896	30,416	1.11 (1.06, 1.16)	74,949	16,312	1.06 (1.01, 1.12)	38,681	7,864	1.00 (0.94, 1.08)
	URINARY	86,578	16,034	1.06 (1.02, 1.11)	46,076	8,185	1.02 (0.97, 1.07)	23,783	3,852	0.95 (0.98, 1.02)
	FEMGEN	122,576	19,527	1.12 (1.03, 1.21)	85,246	13,476	1.05 (0.99, 1.13)	54,335	8,567	0.99 (0.91, 1.10)
	MALEGEN	144,313	11,207	0.83 (0.79, 0.87)	57,834	3,249	0.79 (0.75, 0.84)	13,627	484	0.78 (0.70, 0.88)
	DIGOTHR	21,007	3,123	1.08 (1.03, 1.14)	10,152	1,539	1.03 (0.96, 1.10)	4,864	724	1.00 (0.91, 1.10)
	OTHER	213,200	18,218	0.94 (0.90, 0.98)	124,478	10,063	0.87 (0.83, 0.92)	72,668	5,400	0.81 (0.75, 0.87)
Surgery	No	177,140	19,437	Reference	78,577	8,023	Reference	33,840	3,385	Reference
	Yes	976,002	130,897	0.90 (0.87, 0.94)	568,238	73,680	0.91 (0.88, 0.95)	305,451	38,138	0.90 (0.86, 0.95)
	Unknown	7,361	1,498	1.01 (0.95, 1.07)	5,214	929	1.01 (0.94, 1.09)	3,898	555	1.00 (0.91, 1.10)
Radiotherapy	No	936,411	129,347	Reference	526,478	69,621	Reference	280,841	35,211	Reference
	Yes	223,221	22,353	0.99 (0.97, 1.02)	125,034	12,932	1.03 (1.00, 1.06)	62,059	6,814	1.06 (1.03, 1.10)
	Unknown	871	132	1.23 (1.03, 1.46)	517	79	1.23 (0.98, 1.54)	289	53	1.43 (1.09, 1.87)
Chemotherapy	None/Unknown	978,119	139,567	Reference	554,562	76,168	Reference	294,774	38,940	Reference
	Yes	182,384	12,265	0.85 (0.81, 0.90)	97,467	6,464	0.88 (0.85, 0.91)	48,415	3,138	0.90 (0.87, 0.94)
SEER historic stage	*In situ*	128,461	12,864	Reference	72,907	7,101	Reference	36,890	3,534	Reference
	Distant	47,601	4,826	0.92 (0.83, 1.03)	25,260	2,467	0.98 (0.91, 1.06)	13,677	1,138	0.97 (0.90, 1.05)
	Localized/Regional	877,458	122,427	1.07 (1.04, 1.10)	494,097	66,742	1.08 (1.05, 1.11)	258,074	34,010	1.10 (1.06, 1.14)
	Unknown	106,983	11,715	1.08 (1.02, 1.15)	59,765	6,322	1.12 (1.07, 1.18)	34,548	3,396	1.16 (1.09, 1.22)

*Marital status at diagnosis, race, calendar years at diagnosis according to 1970, 1980, 1990, 2000, 2010, nine original registries, and all variables listed in the table were adjusted in the multivariate competing risk regression models. HR (95%CI), Hazard ratio (95% confidence intervals); RESPIR, respiratory; BREAST, breast; COLRECT, colon and rectum; URINARY, urinary; LYMYLEUK, lymphoma of all sites and leukemia; FEMGEN, female genital; MALEGEN, male genital; DIGOTHR, other digestive; OTHER, all other sites.

## Discussion

In this cohort of more than 4.8 million patients with cancer, the risk of CV death begins to climb after a cancer diagnosis and will eventually outweigh the risk of cancer death for most patients with cancer. Specifically, it took 15 years for P_CV_ and 25 years for CMR_CV_ in the aggregate of patients to outweigh P_CA_ and CMR_CA_. We also observed that older age at diagnosis was the most important factor associated with the increased risk of CV death in patients with cancer. Moreover, as reported in recently published studies, the overall CV mortality rates for the general population in the USA were 252.7 deaths and 219.4 deaths per 100,000 person-years in 2014 (31) and 2017 (32), respectively. For the population older than 65 years in the USA, the CV mortality rate was 1,407.2 deaths per 100,000 person-years. In this study, the overall CV mortality rate was 16.99 deaths/1,000 person-years (namely, 1,699 deaths per 100,000 person-years), which was much higher in older patients with cancer. These results indirectly supported the higher CV mortality in patients with cancer than that in the general population.

Previous studies suggested that CV death in patients with cancer can be primarily attributable to CV toxicity associated with various anticancer treatments, including chemotherapy, radiotherapy, endocrine therapy (10, 33–35), and targeted therapy (i.e., trastuzumab) (36, 37). Patients with cancer are also potentially at high CV risk when undergoing surgery. This would be due to the traumatic stress on the CV system caused by surgery and chronic inflammation after surgery (4, 13). An increased risk of stress-induced CV death caused by surgery was usually an immediate risk but not a long-term risk (4). The difference between this study and previous studies is that chemotherapy and surgery do not significantly increase the risk of CV death but reduce the risk in 10-year survivors. Even though long-term cancer survivors were selected to minimize the dilution effect of a short-term follow-up, a reduced risk of CV death associated with chemotherapy was still observed, even further adjusting the main confounding factors, such as age at diagnosis, cancer stage, and competing risk of death. Due to the non-randomized design within this study, other unknown factors may also contribute to a reduced risk of CV death, such as potentially higher exposure to risk factors for CVD for patients who do not receive chemotherapy than for those who can receive chemotherapy. Therefore, further studies with a more sophisticated design are needed in the future to validate the results. These results also suggested that the combined effect of chemotherapy on CV death for patients with cancer should be re-evaluated in addition to well-established CV toxicity. A similar re-evaluation is also needed for a reduced risk of CV death associated with surgery for patients with cancer. Unlike a reduced risk of CV death associated with both chemotherapy and surgery, the combined effect of radiotherapy changed from no effect to a significantly increased risk of CV death (Table 2). The results suggest that cardio-oncologists should pay more attention to the CV toxicity of radiotherapy than to chemotherapy.

Compared to the associations between the risk of CV death and other factors, this study suggested that the most important risk factor for CV death was older age at diagnosis. The risk of CV death was even 40 times higher in patients with cancer diagnosed at age ≥ 80 years compared to those diagnosed at age ≤ 30 years. This huge difference is similar to the results of previous studies (13, 38, 39). This is probably due to the presence of shared risk factors and chronic inflammation between CVD and cancer (39). However, this huge difference has not received enough attention up to now. These results also suggested that integrated management of shared risk factors would be an urgent need to reduce the overall burden of multimorbidity among long-time cancer survivors.

Another important finding of this study is that the timing of CV death over cancer death varies among patients at different sites of cancer. It first appeared in patients with colorectal cancer and was last observed in patients with breast cancer. However, we also found that patients with cancer not at all sites would experience more CV deaths than cancer deaths. This is likely to be related to the different treatment regimens of patients with different sites of cancer. It may also be related to the potency of local/distant metastases for cancers at different sites. Distant metastasis is based on vascular invasion. Patients with cancer may be in a state where the CV system continuously faces the invasion process by cancer cells and inflammatory factors. Cancer patients who are more prone to distant metastasis are more prone to CV invasion (40–42). Therefore, prevention of distant metastasis in patients with cancer may be an effective way to reduce the risk of CV death.

Several limitations deserved attention in this study. First, preexisting illnesses, especially preexisting CVD, may modify the risk of CV death after a cancer diagnosis. Second, shared risk factors between cancer and CVD, such as residual confounding, may bias our findings. Third, as the data were collected from cancer registries rather than CV registries, underreporting or misclassification of CVD would also bias our results. Fourth, different treatment components may be associated with different CV mortality risks. However, the SEER data did not provide more detailed information to reclassify patients who received radiotherapy into subgroups with chest or non-chest radiotherapy, subgroups with right- or left-side chest radiotherapy, or other subgroups. Another potential limitation would be selection bias for all-cause mortality due to better health conditions of long-term survivors compared to short-term survivors. Due to the exclusion of patients who died from any cause within a specific time point (e.g., 10 years), including those who died from CV death within that time point, this selection bias would dilute the overall risk of CV death in patients with cancer. Finally, as there was no available information in SEER to analyze age-standardized CV mortality in the general population, we cannot directly calculate the relative risk of CV mortality in patients with cancer compared to those in the general population.

Despite the limitations of this study mentioned earlier, such a large real-world cohort study with relatively reasonable analyses would provide many insights into the current practice of cardio-oncology. All these limitations will be our next focus in the future. Additionally, although we observed higher CVD-specific mortality than the index cancer-specific mortality among long-term cancer survivors, we recommend focusing more on cardio-oncology care than cardiotoxic therapy for long-term cancer survivors, as they would not normally be exposed in long-term exposure to cardiotoxic therapy after receiving standard anticancer therapies.

In conclusion, for patients with cancer, the risk of CV death persists throughout life and is likely to exceed the risk of cancer death over time. The risk of CV death in elderly patients with cancer and radiotherapy, as well as the prevention of CV death by reducing local/distant metastases, will be the focus in the field of cardio-oncology.

## Data availability statement

The original contributions presented in this study are included in the article/Supplementary material, further inquiries can be directed to the corresponding authors.

## Ethics statement

Ethical review and approval was not required for this study in accordance with the local legislation and institutional requirements.

## Author contributions

All authors involved with the conception and design, manuscript writing, and final approval of the manuscript.

## Supplementary material

Supplementary Figure 1 Competing mortality risk curves for patients with cancer who survived for more than 10 years by cancer sites. CVD, cardiovascular disease; RESPIR, respiratory; BREAST, breast; COLRECT, colon and rectum; URINARY, urinary; LYMYLEUK, lymphoma of all sites and leukemia; FEMGEN, female genital; MALEGEN, male genital; DIGOTHR, other digestive; OTHER, all other sites.

Supplementary Figure 2 Competing mortality risk curves for patients with cancer who survived for more than 15 years by cancer sites. CVD, cardiovascular disease; RESPIR, respiratory; BREAST, breast; COLRECT, colon and rectum; URINARY, urinary; LYMYLEUK, lymphoma of all sites and leukemia; FEMGEN, female genital; MALEGEN, male genital; DIGOTHR, other digestive; OTHER, all other sites.

Supplementary Figure 3 Competing mortality risk curves for selected patients with cancer who survived for more than 25 and 30 years. RESPIR, respiratory; LYMYLEUK, lymphoma of all sites and leukemia.
