# Supplementary material for: Higher risk of cardiovascular mortality than cancer mortality among long-term cancer survivors
Source: Front Cardiovasc Med. 2023 Jan 25;10:1014400. doi: 10.3389/fcvm.2023.1014400 (PMC9905625; doi:10.3389/fcvm.2023.1014400)
Supplement: Supplementary file 1 [file Table_1.docx]

**Supplementary table S1. Cardiovascular mortality risk and age at diagnosis in long-term cancer survivors*.**

| Cancer site | Variable | Groups | Cancer patients survived ≥ 20 years | | |
| --- | --- | --- | --- | --- | --- |
|  |  |  | Number of | Number of | HR (95%CI) |
|  |  |  | subgroups | cardiovascular death |  |
| LYMYLEUK | Age at | ≤ 30 | 10384 | 338 | Reference |
|  | diagnosis, | 31-39 | 3896 | 210 | 1.73 (1.42, 2.11) |
|  | years | 41-49 | 3670 | 253 | 2.50 (2.05, 3.05) |
|  |  | 51-59 | 3633 | 528 | 5.16 (4.31, 6.17) |
|  |  | 61-69 | 2353 | 522 | 9.72 (8.12, 11.60) |
|  |  | 71-77 | 436 | 139 | 17.80 (14.00, 22.70) |
|  |  | ≥ 80 | 9 | 5 | 32.80 (10.60, 102.00) |
| RESPIR | Age at | ≤ 30 | 495 | 11 | Reference |
|  | diagnosis, | 31-39 | 654 | 53 | 3.73 (1.90, 7.31) |
|  | years | 41-49 | 1497 | 150 | 4.66 (2.45, 8.88) |
|  |  | 51-59 | 2290 | 368 | 7.37 (3.89, 13.94) |
|  |  | 61-69 | 1481 | 284 | 10.73 (5.66, 30.34) |
|  |  | 71-77 | 224 | 58 | 18.33 (9.21, 36.45) |
|  |  | ≥ 80 | 5 | 0 | - |
| BREAST | Age at | ≤ 30 | 3750 | 95 | Reference |
|  | diagnosis, | 31-39 | 13592 | 471 | 1.40 (1.13, 1.74) |
|  | years | 41-49 | 31626 | 1780 | 2.45 (2.00, 3.00) |
|  |  | 51-59 | 29241 | 3682 | 5.55 (4.53, 6.78) |
|  |  | 61-69 | 20899 | 4627 | 11.76 (9.62, 14.38) |
|  |  | 71-77 | 4871 | 1521 | 20.20 (16.42, 24.86) |
|  |  | ≥ 80 | 225 | 92 | 31.17 (22.82, 42.57) |
| COLRECT | Age at | ≤ 30 | 952 | 31 | Reference |
|  | diagnosis, | 31-39 | 2516 | 135 | 1.76 (1.20, 2.58) |
|  | years | 41-49 | 6556 | 680 | 3.49 (2.45, 4.98) |
|  |  | 51-59 | 12374 | 2185 | 6.00 (4.23, 8.52) |
|  |  | 61-69 | 12659 | 3473 | 10.87 (7.66, 15.41) |
|  |  | 71-77 | 3447 | 1267 | 17.30 (12.16, 24.63) |
|  |  | ≥ 80 | 177 | 93 | 30.36 (19.90, 46.32) |
| URINARY | Age at | ≤ 30 | 1902 | 43 | Reference |
|  | diagnosis, | 31-39 | 2326 | 117 | 2.13 (1.47, 3.08) |
|  | years | 41-49 | 4955 | 512 | 4.56 (3.26, 6.37) |
|  |  | 51-59 | 7250 | 1253 | 7.86 (5.65, 10.92) |
|  |  | 61-69 | 6068 | 1496 | 13.25 (9.54, 18.42) |
|  |  | 71-77 | 1222 | 405 | 21.53 (15.30, 30.29) |
|  |  | ≥ 80 | 60 | 26 | 33.22 (19.66, 56.11) |
| FEMGEN | Age at | ≤ 30 | 6350 | 106 | Reference |
|  | diagnosis, | 31-39 | 9157 | 418 | 2.87 (2.32, 3.55) |
|  | years | 41-49 | 11755 | 1199 | 6.30 (5.16, 7.70) |
|  |  | 51-59 | 15061 | 3064 | 11.00 (9.04, 13.38) |
|  |  | 61-69 | 10076 | 3078 | 20.77 (17.07, 25.29) |
|  |  | 71-77 | 1883 | 675 | 32.14 (26.01, 39.72) |
|  |  | ≥ 80 | 53 | 27 | 51.52 (31.74, 83.63) |
| MALEGEN | Age at | ≤ 30 | 4829 | 100 | Reference |
|  | diagnosis, | 31-39 | 3287 | 105 | 1.90 (1.41, 2.55) |
|  | years | 41-49 | 1619 | 87 | 3.92 (2.84, 5.39) |
|  |  | 51-59 | 1710 | 85 | 7.66 (5.52, 10.62) |
|  |  | 61-69 | 1805 | 88 | 20.77 (14.59, 29.56) |
|  |  | 71-77 | 376 | 19 | 31.05 (16.92, 56.96) |
|  |  | ≥ 80 | 1 | 0 | - |
| DIGOTHR | Age at | ≤ 30 | 553 | 17 | Reference |
|  | diagnosis, | 31-39 | 600 | 30 | 1.83 (0.99, 3.36) |
|  | years | 41-49 | 1040 | 97 | 3.45 (2.00, 5.95) |
|  |  | 51-59 | 1409 | 242 | 6.47 (3.85, 10.89) |
|  |  | 61-69 | 1002 | 249 | 10.04 (6.17, 17.65) |
|  |  | 71-77 | 253 | 88 | 16.90 (9.63, 29.82) |
|  |  | ≥ 80 | 7 | 1 | 9.98 (1.17, 84.80) |
| OTHER | Age at | ≤ 30 | 18938 | 242 | Reference |
|  | diagnosis, | 31-39 | 16319 | 405 | 2.40 (2.03, 2.84) |
|  | years | 41-49 | 15882 | 918 | 5.96 (5.11, 6.96) |
|  |  | 51-59 | 12768 | 1691 | 13.34 (11.49, 15.49) |
|  |  | 61-69 | 7276 | 1677 | 29.54 (25.38, 34.37) |
|  |  | 71-77 | 1425 | 454 | 53.53 (44.82, 63.93) |
|  |  | ≥ 80 | 60 | 13 | 34.75 (18.66, 64.71) |

*, marital status at diagnosis, race, calendar years at diagnosis according to 1970s, 1980s, 1990s, 2000s, 2010s, nine original registries, sex, SEER historic stage, surgery, radiotherapy and chemotherapy were adjusted in the adjusted in the multivariate competing risk regression models. Note: HR (95%CI), Hazard ratio (95% confidence intervals); RESPIR, respiratory; BREAST, breast; COLRECT, colon and rectum; URINARY, urinary; LYMYLEUK, lymphoma of all sites and leukemia; FEMGEN, female genital; MALEGEN, male genital; DIGOTHR, other digestive; OTHER, all other sites.
