# Supplementary figures and images for: Higher risk of cardiovascular mortality than cancer mortality among long-term cancer survivors
Source: Front Cardiovasc Med. 2023 Jan 25;10:1014400. doi: 10.3389/fcvm.2023.1014400 (PMC9905625; doi:10.3389/fcvm.2023.1014400)

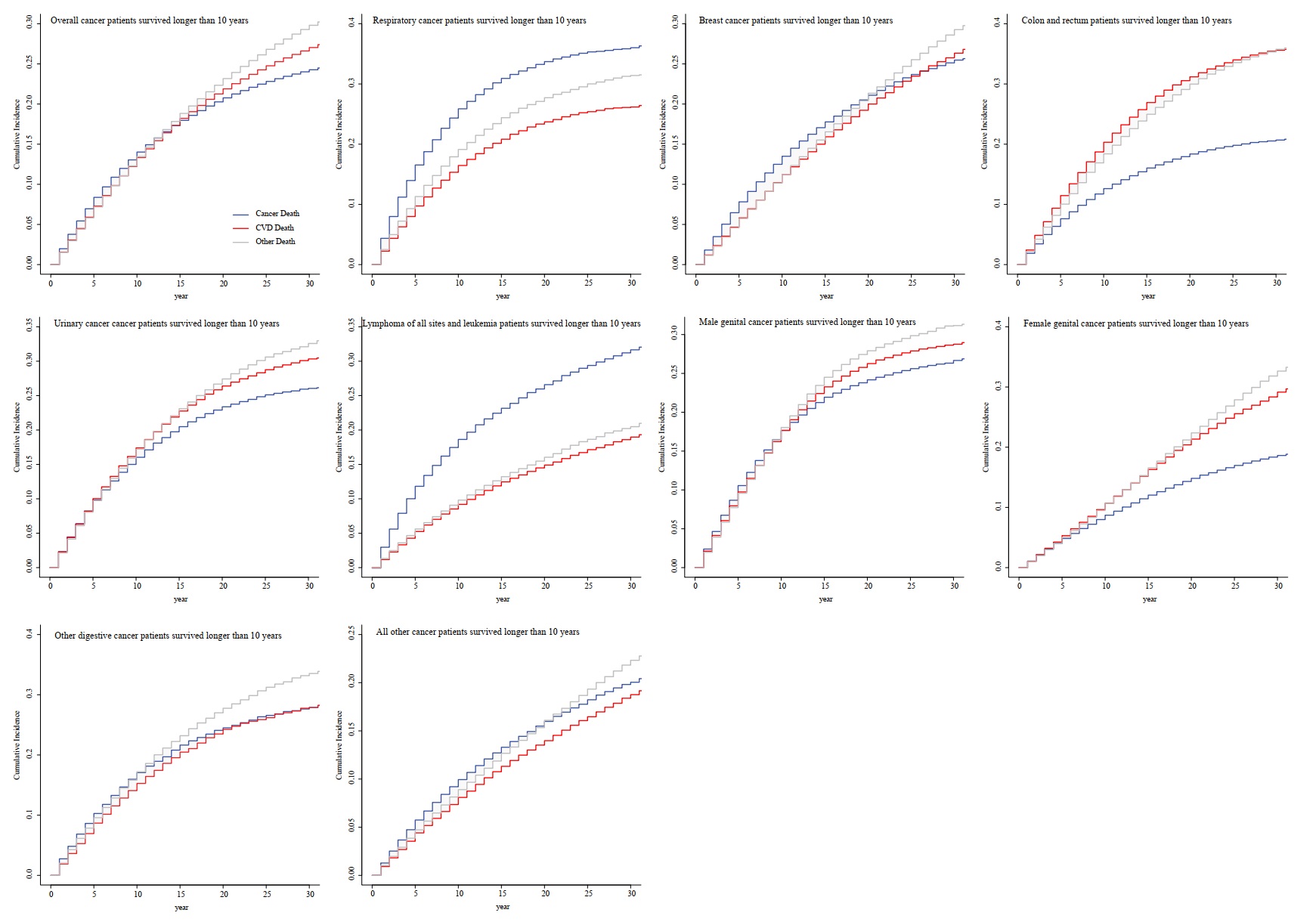

Supplement: Supplementary Figure 1 — Competing mortality risk curves for patients with cancer who survived for more than 10 years by cancer sites. CVD, cardiovascular disease; RESPIR, respiratory; BREAST, breast; COLRECT, colon and rectum; URINARY, urinary; LYMYLEUK, lymphoma of all sites and leukemia; FEMGEN, female genital; MALEGEN, male genital; DIGOTHR, other digestive; OTHER, all other sites. [file Image_1.JPEG]

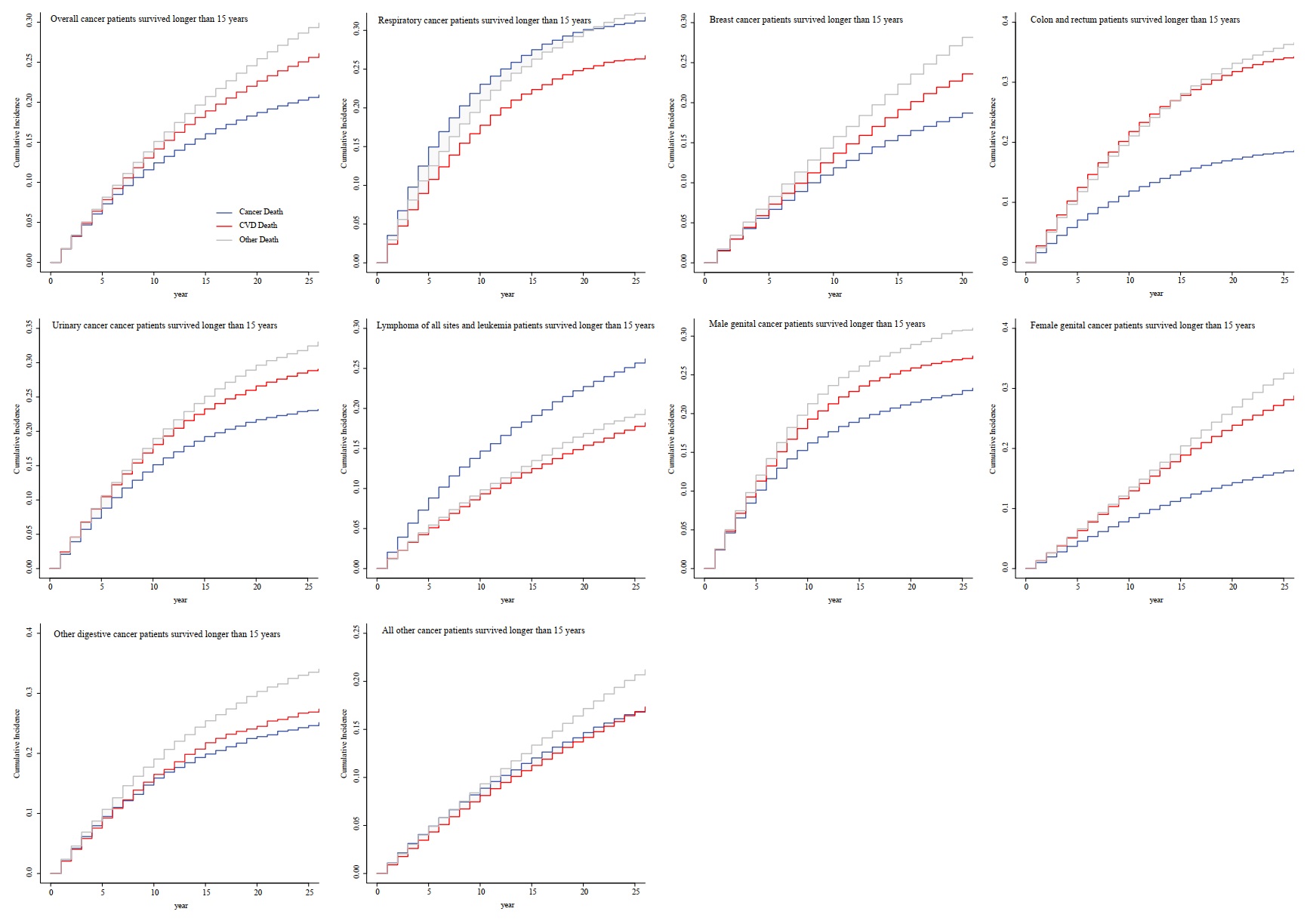

Supplement: Supplementary Figure 2 — Competing mortality risk curves for patients with cancer who survived for more than 15 years by cancer sites. CVD, cardiovascular disease; RESPIR, respiratory; BREAST, breast; COLRECT, colon and rectum; URINARY, urinary; LYMYLEUK, lymphoma of all sites and leukemia; FEMGEN, female genital; MALEGEN, male genital; DIGOTHR, other digestive; OTHER, all other sites. [file Image_2.JPEG]

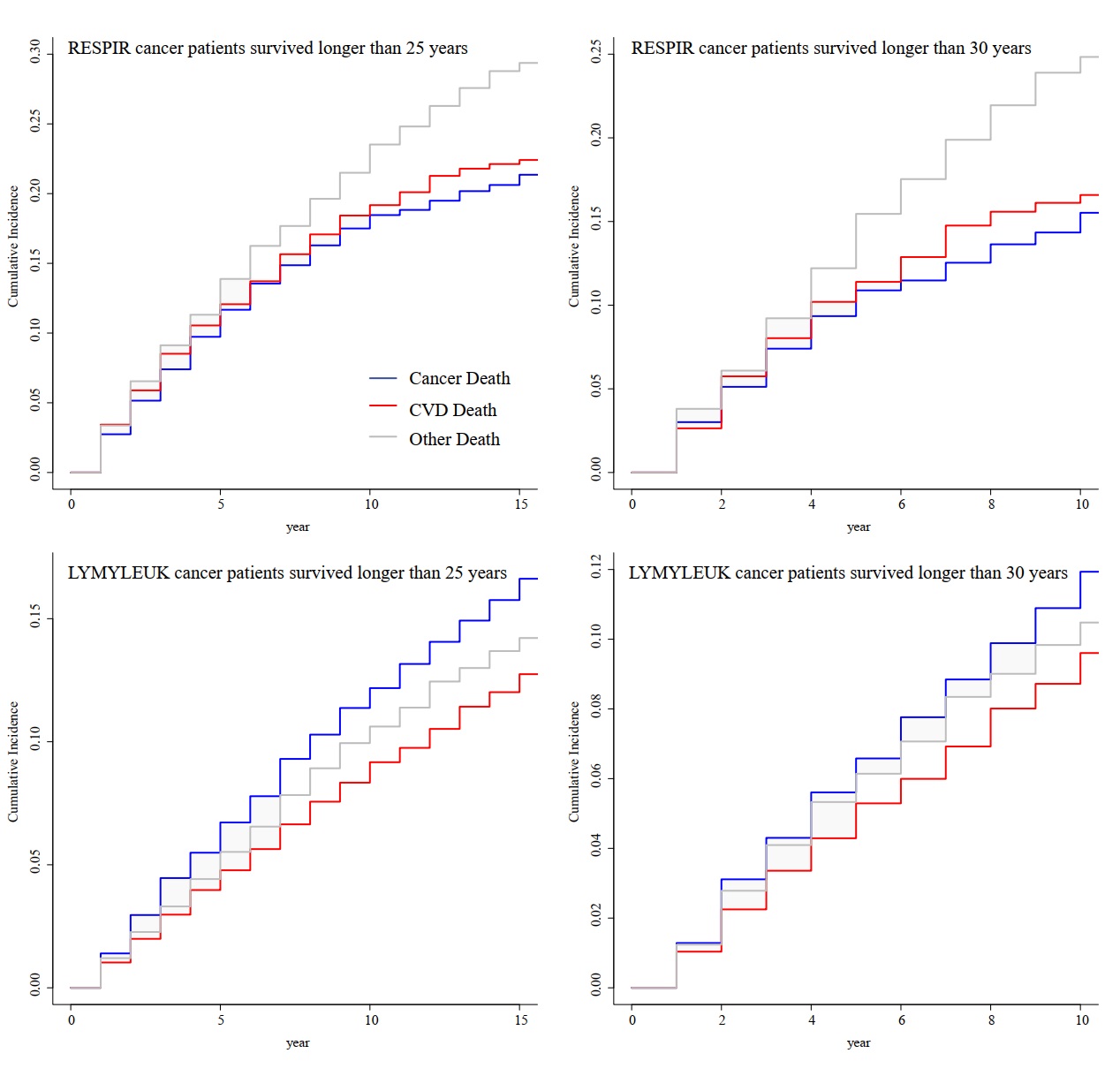

Supplement: Supplementary Figure 3 — Competing mortality risk curves for selected patients with cancer who survived for more than 25 and 30 years. RESPIR, respiratory; LYMYLEUK, lymphoma of all sites and leukemia. [file Image_3.JPEG]
